# Effects of Fatty Acid Treatments on the Dexamethasone-Induced Intramuscular Lipid Accumulation in Chickens

**DOI:** 10.1371/journal.pone.0036663

**Published:** 2012-05-18

**Authors:** Xiao juan Wang, Dai lin Wei, Zhi gang Song, Hong chao Jiao, Hai Lin

**Affiliations:** 1 Department of Animal Science, Shandong Agricultural University, Taian, Shandong, People’s Republic of China; 2 Department of Endocrine, Taian Central Hospital, Taian, Shandong, People’s Republic of China; Institut Pluridisciplinaire Hubert Curien, France

## Abstract

**Background:**

Glucocorticoid has an important effect on lipid metabolism in muscles, and the type of fatty acid likely affects mitochondrial utilization. Therefore, we hypothesize that the different fatty acid types treatment may affect the glucocorticoid induction of intramuscular lipid accumulation.

**Methodology/Principal Findings:**

The effect of dexamethasone (DEX) on fatty acid metabolism and storage in skeletal muscle of broiler chickens (*Gallus gallus domesticus*) was investigated with and without fatty acid treatments. Male Arbor Acres chickens (31 d old) were treated with either palmitic acid (PA) or oleic acid (OA) for 7 days, followed by DEX administration for 3 days (35–37 d old). The DEX-induced lipid uptake and oxidation imbalance, which was estimated by increased fatty acid transport protein 1 (FATP1) expression and decreased carnitine palmitoyl transferase 1 activity, contributed to skeletal muscle lipid accumulation. More sensitive than glycolytic muscle, the oxidative muscle in DEX-treated chickens showed a decrease in the AMP to ATP ratio, a decrease in AMP-activated protein kinase (AMPK) alpha phosphorylation and its activity, as well as an increase in the phosphorylation of mammalian target of rapamycin (mTOR) and ribosomal p70S6 kinase, without Akt activation. DEX-stimulated lipid deposition was augmented by PA, but alleviated by OA, in response to pathways that were regulated differently, including AMPK, mTOR and FATP1.

**Conclusions:**

DEX-induced intramuscular lipid accumulation was aggravated by SFA but alleviated by unsaturated fatty acid. The suppressed AMPK and augmented mTOR signaling pathways were involved in glucocortcoid-mediated enhanced intramuscular fat accumulation.

## Introduction

Fatty acids and glucose are the primary skeletal muscle fuels. Excessive glucocorticoid (GC) has been correlated with a predisposition to many metabolic syndromes, including dyslipidemia and insulin resistance [1]. GC has an important effect on lipid metabolism in muscles. GC and insulin act together to promote intramyocellular lipid accumulation in mammals [2] and chickens [3]. Compared with mammals, birds have higher level of glucose and lower concentration of insulin [4], [5], and have more refractory insulin cascade in skeletal muscle tissues [4], [6]. Increased blood lipid flux and mismatched lipid uptake and oxidation are suggested to be responsible for intramyocellular lipid accumulation in chickens [3], [7], [8]. The study on chicken model would be helpful to the understanding of intramyocellular lipid accumulation.

The type of fatty acid likely affects mitochondrial utilization. Myotube mitochondrial activity is increased with unsaturated fatty acid (UFA, [9]) but is impaired by SFA [9], [10]. Whether imbalanced fatty acid uptake and utilization induced by GC is influenced by different types of fatty acids remains unclear.

In mammals, AMP-activated protein kinase (AMPK) is involved in cellular energy homeostasis regulation. AMPK, which is thought to be a positive regulator of intracellular fatty acid metabolism, is activated by an increase in the AMP to ATP ratio [11]. AMPK phosphorylates and inactivates acetyl-CoA carboxylase (ACC) and, in turn, diminishes malonyl-CoA; thus, carnitine palmitoyl transferase 1 (CPT1) inhibition is relieved, and free fatty acid entry into the mitochondria is facilitated for β-oxidation [12]. Therefore, excessive lipid accumulation in the skeletal muscle in obesity could be from the dysregulation of the AMPK/malonyl-CoA fuel-sensing system [13]. AMPK activation stimulates fatty acid oxidation in the skeletal muscle by activating PPARs [14]. The functional liver kinase B1 (LKB1)/AMPK pathway in chickens is similar to the pathway in mammals [15].

Mammalian target of rapamycin (mTOR) is a serine/threonine protein kinase that has been proposed to play a central role in skeletal muscle nutrient and energy sensing, by phosphorylating two downstream proteins, eukaryotic initiation factor 4E-binding protein 1 (4E-BP1) and ribosomal p70S6 kinase (p70S6K)1 [16]. Porstmann et al. [17] demonstrated that the expression of enzymes involved in lipid biosynthesis, such as ACC, was regulated by the Akt/mTOR signaling network. Furthermore, mTOR inhibition in rat skeletal muscle cells improved fatty acid oxidation [18]. Excessive lipid availability and AMPK are involved in mTOR regulation. Lipid-induced mTOR activation and insulin resistance in rat skeletal muscle were reversed by improved muscle AMPK activation [19]. In mammalian skeletal muscle cells, GC could abrogate mTOR signaling by dephosphorylating p70S6K and 4E-BP1 [20]. Therefore, we hypothesize that the different fatty acid types treatment (FA) may affect the GC induction of intramuscular lipid accumulation, which involves the AMPK and mTOR signaling pathways.

Herein, fast growing broiler chickens with high muscle yield and feed efficiency were used as a model for muscle development [21]. Two types of skeletal muscle tissues, oxidative and glycolytic muscles, were investigated to determine tissue specificity in the regulation of lipid metabolism. Dexamethasone (DEX), a synthetic GC that is specific for the GC receptor and delayed plasma clearance [22], was employed to induce the hyperglucocorticoid milieu [3]. Our results indicate that DEX-stimulated lipid deposition was augmented by SFA, but alleviated by UFA. The suppressed AMPK and augmented mTOR signaling pathways were involved in GC-mediated enhanced intramuscular fat accumulation. These findings allow a better view on the metabolic perturbations associated with long-term GC use and dietetical therapy in clinical setting, in a different biological system.

## Materials and Methods

### Ethics Statement

All animal experiments were reviewed and approved by the Institutional Animal Care and Use Committee of Shandong Agricultural University (No. 2001002, Figure S1) and performed in accordance with the “Guidelines for Experimental Animals” of the Ministry of Science and Technology (Beijing, China). All surgery was performed according to Recommendations proposed by European Commission (1997), and all efforts were made to minimize suffering.

### Birds and Husbandry

Male broiler chicks (Arbor Acres, *Gallus gallus domesticus*) were obtained from a local hatchery at 1 d of age and were reared in an environmentally controlled room. The brooding temperature was maintained at 35°C (65% RH) for the first 2 days, then decreased gradually to 21°C (45% RH) until day 28 and was, thereafter, maintained through the end of the experiment (day 38). The light regime was 23L: 1D. All chickens received a starter diet with 21.5% crude protein and 12.37 MJ/kg of metabolisable energy until day 21, after which they received a grower diet with 19.5% crude protein and 12.90 MJ/kg of metabolisable energy [23]. All birds had free access to feed and water during the rearing period.

### Treatment

At 31 d of age, 144 broilers with similar body mass (BM) were allocated to 9 groups, and each group had 16 chickens. The chickens were randomly subjected to one of the following 3 treatments for 7 days: oral infusion of palmitic acid (51.3 mg/kg BM, PA), oleic acid (56.5 mg/kg BM, OA) dissolved with Tween80, and sham infusion of a vehicle-Tween80 (PLA). At 35 d of age, half of the chickens from each group were either randomly exposed to subcutaneous injection of DEX (2 mg/kg BM per day, DEX, [3]) twice per day or sham treated with vehicle-saline (NORM) for 3 days. The use of Tween80 has been validated in previous experiments with regard to lipid metabolism [24], [25]. BM and feed intake were recorded daily.

At 38 d of age, 4 chickens with similar BM (1436±353 g) were selected from each pen. After a 12-h feed withdrawal, a blood sample was drawn from a wing vein using a heparinized syringe within 30 s and collected in iced tubes. Plasma was obtained after centrifugation at 400 g for 10 min at 4°C and was stored at −20°C for further analysis. Immediately after the blood sample was obtained, chickens were killed by cervical dislocation, following exsanguination [26], and then the liver, abdominal fat, subcutaneous fat in the cervical and thigh regions and breast and thigh muscle were harvested and weighed. A 1 to 2 g muscle tissue sample was, respectively, obtained from the left PM (*M. pectoralis major*, fast-twitch glycolytic fiber type muscle) and BF (*M. biceps femoris*, slow-twitch oxidative fiber type muscle), cooled down in liquid nitrogen and stored at −70°C for further analysis.

### Plasma Parameters

The concentrations of glucose (No. F006) and TG (No. F001) in plasma were measured spectrophotometrically using commercial diagnostic kits (Jiancheng, Nanjing, China). The concentration of VLDL was determined using the method described by Griffin and Whitehead [27].

The insulin in plasma was measured using a radioimmunoassay with guinea pig anti-porcine insulin serum (3V, Weifang, China). In this measurement, ^125^I-labeled porcine insulin competes with chicken insulin for sites on an insulin-porcine antibody that is immobilized to the wall of a polypropylene tube. A large cross-reaction has been observed between chicken insulin and the guinea pig anti-porcine sera [28]. The insulin herein is referred to as immunoreactive insulin.

### Intramyocellular Lipid Accumulation

The accumulation of cytoplasmic lipid droplets was visualized by Oil Red O staining according to Lillie and Fullmer [29]. Briefly, frozen tissues were cut in a Leica CM-1850 cryostat microtome (Leica, Wetzlar, Germany). Sixteen- micrometer-thick sections were fixed in formaldehyde and stained with filtered 0.5% Oil Red O (Sigma-Aldrich, St Louis, MO, USA). Morphometric analysis was performed on 10 fields containing transverse muscle fiber sections that were chosen at random for each chicken’s muscles and photographed under an Olympus CX-41 phase-contrast microscope (Olympus, Tokyo, Japan). The volume density of each Oil Red O positive fiber within the muscle tissue was determined by Weibel’s point-counting method [30].

### ATP and AMP Content

The concentrations of ATP and AMP in skeletal muscle tissue were measured with a Waters 515 reversed-phase high performance liquid chromatography system (Waters, Milford, MA, USA) using a modification of the approach described by Ryder [31] and Smolenski and Yacoub [32]. Separation was performed with a reversed-phase Diamonsil C18 column (250 mm×4.6 mm, Dikma, Beijing, China) that was equilibrated with methyl alcohol at room temperature. The injection volume was 10 µL, and flow was maintained at 1 mL/min. Detection of ATP and AMP was achieved at 254 nm with a Waters 2487 Dual λ Absorbance Detector (Waters, Milford, MA, USA) at room temperature. The results were quantitated with external standards (Sigma-Aldrich, St Louis, MO, USA).

### AMPK and CPT1 Activities

AMPK activity was measured by detecting ^32^P release from [^32^P]ATP(5µCi/assay) and with SAMS peptide (His-Met-Arg-Ser-Ala-Met-Ser-Gly-Leu- His-Leu-Val-Lys-Arg-Arg) as the ACC target analog as previous described [33], [34]. The incorporated radioactivity was measured in a SN-6930 scintillation counter (Rihuan, Shanghai, China). The protein concentration was determined using a protein assay kit (Jiancheng, Nanjing, China).

CPT1 activity was defined as 1nmol of CoA-SH released from 1mg of muscle tissue protein per minute according to the method described by Bieber et al. [35]. Protein concentration was determined using a protein assay kit (Jiancheng, Nanjing, China).

### RNA Preparation and Analysis

Gene expression was measured using real-time RT-PCR. Briefly, total RNA from PM and BF was extracted using TRIzol (Invitrogen, San Diego, CA, USA). The quantity and quality of the isolated RNA were determined using a biophotometer (Eppendorf, Hamburg, Germany) and by agarose-gel electrophoresis, respectively. Next, reverse transcription was performed using a RT reaction (10 µL) that consisted of 500 ng total RNA, 5 mmol/L MgCl_2_, 1 µL RT buffer, 1 mmol/L dNTP, 2.5 U AMV, 0.7 nmol/L oligo d(T), and 10 U Ribonuclease inhibitor (TaKaRa, Dalian, China). cDNA was amplified in a 20 µL PCR reaction containing 0.2 µmol/L of each specific primer (Sangon, Shanghai, China) and SYBR green master mix (TaKaRa, Dalian, China). Real-time PCR was performed at 95°C for 10 s of predenaturation, followed by 40 cycles, and each cycle consisted of denaturation at 95°C for 5 s and annealing and extension at 60°C for 40 s. Primers against GAPDH and 18S ribosomal RNA (18S rRNA) were amplified and used as internal controls to normalize the differences in individual samples. The primer sequences for chicken are listed in Table 1. The PCR products were verified by electrophoresis on a 0.8% agarose gel and DNA sequencing. Standard curves were generated using pooled cDNA from the samples that were assayed, and the comparative CT method (2^−ΔΔCT^) was used to quantitate mRNA expression in accordance with Livak and Schmittgen [36]. All of the samples were run in duplicate, and the primers were designed to span an intron to avoid genomic DNA contamination.

**Table 1:** Gene-specific primer of related genes.

Gene	Genebank number	Primers sequences (5′→3′)	Product size
FATP1	DQ352834	Forward tcaggagatgtgttggtgatggat	138 bp
		Reverse cgtctggttgaggatgtgactc	
GAPDH	NM_204305	Forward ctacacacggacacttcaag	244 bp
		Reverse acaaacatgggggcatcag	
INSR	AF111857	Forward caaacggtgaccaagcctca	186 bp
		Reverse catcctgcccatcaaactccg	
L-CPT1	AY675193	Forward ggagaacccaagtgaaagtaatgaa	135 bp
		Reverse gaaacgacataaaggcagaacaga	
M-CPT1	DQ314726	Forward gatttctgctgcttccaattcg	92 bp
		Reverse tgcagcgcgatctgaatg	
PPARα	AF163809	Forward agacaccctttcaccagcatcc	167 bp
		Reverse aacccttacaaccttcacaagca	
18SrRNA	AF173612	Forward ataacgaacgagactctggca	136 bp
		Reverse cggacatctaagggcatcaca	

### Protein Preparation and Western Blot

Proteins were extracted from chicken PM and BF. Muscle samples were homogenized on ice in radioimmunoprecipitation assay buffer (50 mmol/L Tris-HCl at pH 7.4, 1% NP-40, 0.25% sodium deoxycholate, 150 mmol/L NaCl, 1 mmol/L EDTA, 1 mmol/L phenylmethylsulfonyl fluoride, 1 µg/mL aprotinin, 1 µg/mL leupeptin, 1 µg/mL pepstatin, 1 mmol/L sodium orthovanadate, 1 mmol/L sodium fluoride) and centrifuged at 12 000g for 5 minutes at 4°C. Protein concentration was determined using the BCA assay kit (Beyotime, Jiangsu, China). Samples were boiled at 100°C for 5 minutes in 5× sample buffer. Protein extracts (80 µg) were electrophoresed in 7.5–10% SDS polyacrylamide gels (Bio-Rad) according to the Laemmli method [37]. Separated proteins were then transferred onto a nitrocellulose membrane in Tris-glycine buffer containing 20% methanol. Membranes were blocked and immunoblotted with a 1∶1000 dilution of the following primary antibodies: P-AMPKα (Thr172) antibody, AMPKα antibody, P-mTOR (Ser2448) antibody, mTOR antibody, P-p70S6K (Thr389) antibody, and p70S6K antibody. The antibodies were purchased from Cell Signaling Technology (Beverly, MA, USA) and were previously validated for use with chicken samples [15], [38], [39]. The P-Akt (Ser473) antibody and Akt antibody were purchased from Beyotime (Jiangsu, China). Protein was detected using either goat anti-rabbit IgG (H+L)-HRP conjugated secondary antibody (1∶2000, Bio-Rad, Richmond, CA) or HRP-labeled goat anti-mouse IgG (H+L) secondary antibody (1∶1000, Beyotime, Jiangsu, China) with enhanced chemiluminescence (ECL) plus western blot detection reagents (Beyotime, Jiangsu, China). β-actin was used as an internal control (Beyotime, Jiangsu, China). Western blots were quantified using the ImageJ 1.43 software (National Institutes of Health, Bethesda, MD) after the films were scanned.

### Statistical Analysis

A two-way ANOVA model was used to analyze the primary effects of the DEX and FA treatments and their interaction using Statistical Analysis Systems statistical software package (Version 8e, SAS Institute, Cary, NC, USA). Homogeneity of variances among groups was confirmed using Bartlett’s test (SAS Institute). When the primary effect of a treatment was significant, the differences between the means were assessed using Duncan’s multiple range analysis. The mean was considered to be significantly different at *P*<0.05.

## Results

### Animal Growth and Tissue Development

DEX significantly decreased BM gain (*P*<0.0001), whereas FA action was interacted by DEX treatment (*P*<0.01), and the NORM-OA chickens had the highest BM gain. OA increased BM gain in NORM-chickens. After DEX treatment, however, FA had no significant effect on BM gain (Table 2). DEX had no significant effect on feed intake. However, FA tended to have an effect on feed intake (*P* = 0.07). Feed efficiency was significantly decreased by DEX (*P*<0.0001) and not by FA. There was no significant interaction between DEX and FA for either feed intake or feed efficiency (Table 2).

**Table 2:** The effect of DEX (daily subcutaneous injection of 2 mg/kg BM for 3d) and FA treatments (daily gastric infusion of palmitic acid or oleic acid for 7d) on the growing performance of broiler chickens.

Item	Treat	DEX	NORM	Probability
BM gain (g/d)	PA	28.2±0.86 c	42.2±0.27 b	P_DEX_<0.0001
	OA	26.5±1.32 c	51.0±0.99 a	P_FA_ = 0.11
	PLA	31.2±1.86 c	41.5±2.85 b	P_DEX×FA_ = 0.0022
Feed intake (g/d)	PA	99.0±3.84 ab	92.4±6.10 b	P_DEX_ = 0.38
	OA	93.2±3.52 ab	95.8±3.21 ab	P_FA_ = 0.07
	PLA	107±3.60 a	102±4.02 ab	P_DEX×FA_ = 0.51
Feed:gain (g/g)	PA	3.51±0.03 a	2.19±0.14 bc	P_DEX_<0.0001
	OA	3.52±0.09 a	1.88±0.10 c	P_FA_ = 0.30
	PLA	3.45±0.22 a	2.49±0.28 b	P_DEX×FA_ = 0.17

Values are means ± SE (n = 3);

a, b, c: Means within the same item with different letter differ significantly, *P*<0.05.

P_DEX_: P-value of main effect of DEX treatment; P_FA_: P-value of main effect of FA treatment; P_DEX×FA_: P-value of interaction between DEX and FA treatments.

The liver weight was significantly influenced by DEX, FA, and their interaction (*P*<0.0001, Table 3). The DEX-PA chickens had the highest and Control chickens had the lowest. DEX significantly improved the abdominal (*P*<0.0001), cervical (*P*<0.01), and thigh fat masses (*P*<0.0001). In contrast, FA treatment affected significantly only in abdominal fat (*P*<0.01). PA chickens had higher abdominal fat mass compared with either the OA or PLA groups. DEX and FA had no significant interaction in abdominal, cervical, and thigh fat masses. Neither DEX nor FA treatment had a detectable effect on breast and thigh muscle masses (Table 3).

**Table 3:** The effect of DEX (daily subcutaneous injection of 2 mg/kg BM for 3d) and FA treatments (daily gastric infusion of palmitic acid or oleic acid for 7d) on the yield of skeletal muscle and liver and the fat accumulation in adipose tissues (% BM) of broiler chickens.

Item	Treat	DEX	NORM	Probability
Liver	PA	4.08±0.10 a	2.08±0.04 c	P_DEX_<0.0001
	OA	2.84±0.03 b	2.01±0.04 c	P_FA_<0.0001
	PLA	3.03±0.16 b	1.96±0.07 c	P_DEX×FA_<0.0001
Abdominal fat	PA	1.56±0.12 a	1.11±0.15 b	P_DEX_<0.0001
	OA	1.24±0.11 ab	0.661±0.09 c	P_FA_ = 0.0017
	PLA	1.41±0.10 ab	0.558±0.09 c	P_DEX×FA_ = 0.21
Cervical fat	PA	0.348±0.05 ab	0.175±0.03 b	P_DEX_ = 0.0049
	OA	0.353±0.05 ab	0.257±0.05 ab	P_FA_ = 0.70
	PLA	0.377±0.09 a	0.230±0.07 ab	P_DEX×FA_ = 0.80
Thigh fat	PA	0.366±0.03 ab	0.170±0.03 d	P_DEX_<0.0001
	OA	0.322±0.03 bc	0.161±0.03 d	P_FA_ = 0.11
	PLA	0.491±0.10 a	0.196±0.05 cd	P_DEX×FA_ = 0.37
Breast	PA	17.1±0.52	17.1±0.50	P_DEX_ = 0.99
	OA	17.6±0.36	17.1±0.30	P_FA_ = 0.87
	PLA	17.0±0.59	17.4±0.42	P_DEX×FA_ = 0.59
Thigh	PA	13.6±0.26	13.5±0.37	P_DEX_ = 0.28
	OA	13.8±0.29	13.6±0.30	P_FA_ = 0.81
	PLA	13.9±0.27	13.6±0.26	P_DEX×FA_ = 0.96

Values are means ± SE (n = 12);

a, b, c, d: Means within the same item with different superscript differ significantly, *P*<0.05.

P_DEX_: P-value of main effect of DEX treatment; P_FA_: P-value of main effect of FA treatment, P_DEX×FA_: P-value of interaction between DEX and FA treatments.

### Intramuscular Lipid Accumulation

**Figure 1:**
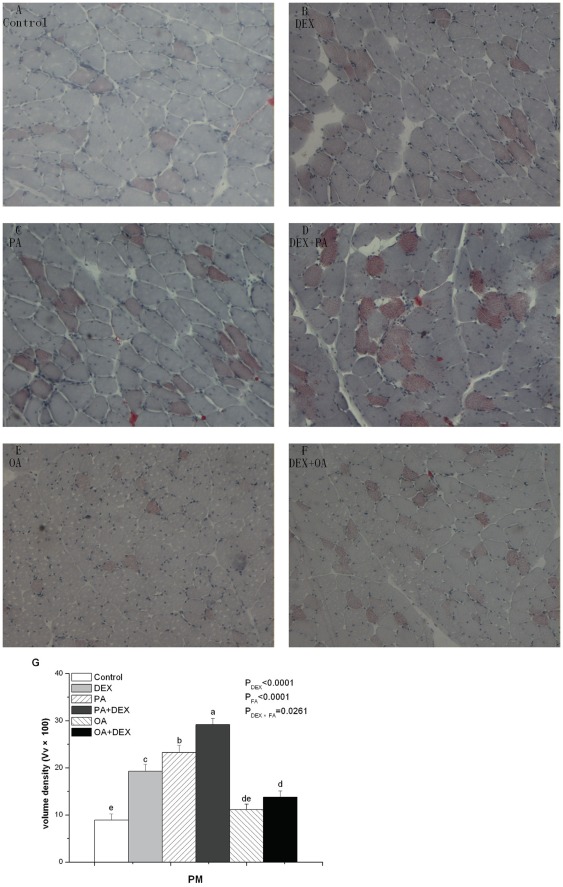
Oil Red O staining of skeletal muscle for cytoplasmic lipid droplets in PM. Figure A–F show the effect of DEX (daily subcutaneous injection of 2 mg/kg body mass for 3d) and FA treatments (daily gastric infusion of either palmitic acid or oleic acid for 7d) and the interaction between DEX and FA on lipid accumulation in the myocytes from PM in broiler chickens. Figure G indicates the volume density (Vv×100) of Oil Red O-positive muscle fibers in skeletal muscle. The values are the means ± SE (n = 8); Means with a different letter differ significantly (*P*<0.05). P_DEX_: P-value of main effect of DEX treatment; P_FA_: P-value of main effect of FA treatment; P_DEX×FA_: P-value of interaction between DEX and FA treatments.

The intramuscular fat (IMF) content in PM was significantly affected by DEX (*P*<0.0001), FA (*P*<0.0001), and their interaction (*P*<0.05, Figure 1). The DEX-PA chickens had the highest and Control chickens had the lowest. After DEX treatment, PA chickens had the highest IMF content, and the OA group had the lowest IMF level. In NORM-chickens, the IMF content was higher in PA chickens than the IMF content in either the OA or PLA group, whereas there was no significant difference between OA and PLA treated chickens.

**Figure 2:**
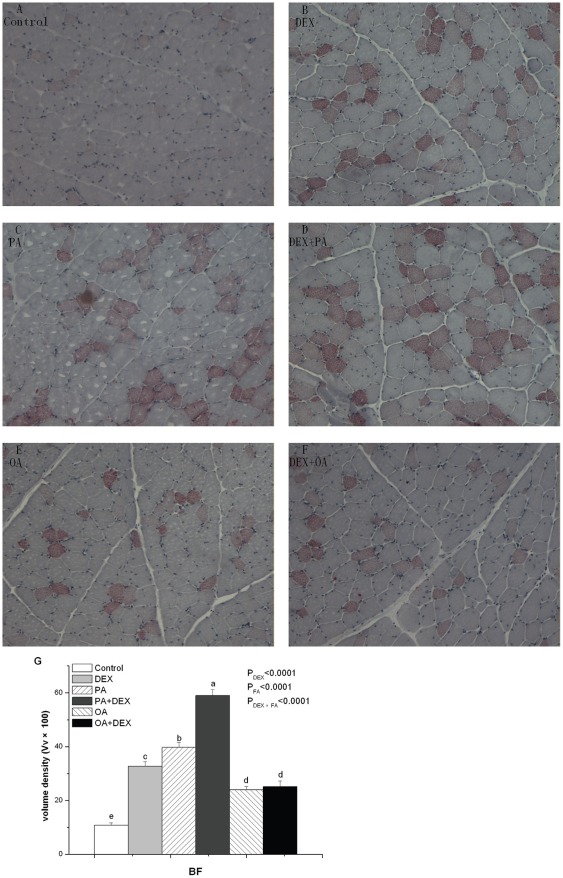
Oil Red O staining of skeletal muscle for cytoplasmic lipid droplets in BF. Figure A–F show the effect of DEX (daily subcutaneous injection of 2 mg/kg body mass for 3d) and FA treatments (daily gastric infusion of either palmitic acid or oleic acid for 7d) and the interaction between DEX and FA on lipid accumulation in myocytes from BF in broiler chickens. Figure G indicates the volume density (Vv×100) of Oil Red O-positive muscle fibers in skeletal muscle. The values are the means ± SE (n = 8); Means with a different letter differ significantly (*P*<0.05). P_DEX_: P-value of main effect of DEX treatment; P_FA_: P-value of main effect of FA treatment; P_DEX×FA_: P-value of interaction between DEX and FA treatments.

In BF, the IMF content was significantly affected by DEX, FA, and their interaction (*P*<0.0001, Figure 2). The DEX-PA chickens had the highest and Control chickens had the lowest. In the DEX treated chickens, the PA chickens had higher and the OA chickens had lower IMF level compared with the DEX-PLA chickens. In the NORM treated chickens, however, the PA group had higher IMF level, while PLA chickens had lower IMF level compared with OA chickens.

### Plasma Parameters

**Figure 3:**
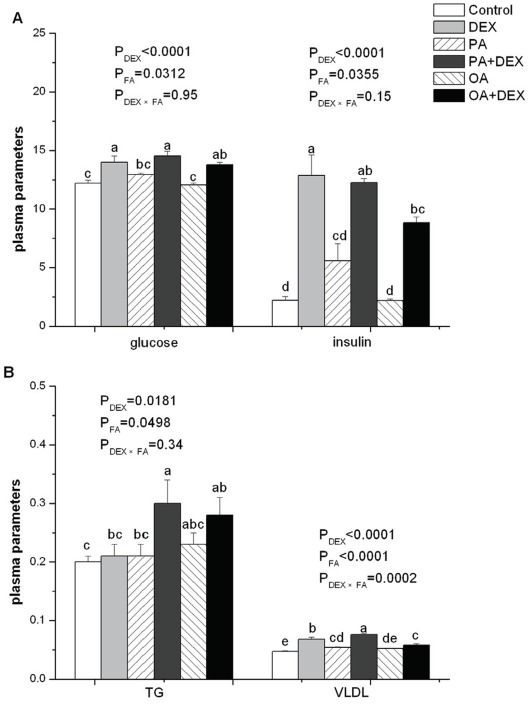
The effect of DEX and FA on plasma parameters. Figure A, B show the effect of DEX (daily subcutaneous injection of 2 mg/kg body mass for 3d) and FA treatments (daily gastric infusion of either palmitic acid or oleic acid for 7d) and the interaction between DEX and FA on the plasma concentration of glucose (mmol/L, A), insulin (µIU/Ml, A), TG (mmol/L, B), and VLDL (Abs, B) in broiler chickens. The values are the means ± SE (n = 12); Means with a different letter differ significantly (*P*<0.05). P_DEX_: P-value of main effect of DEX treatment; P_FA_: P-value of main effect of FA treatment; P_DEX×FA_: P-value of interaction between DEX and FA treatments.

Plasma concentrations of glucose, insulin, TG, and VLDL were significantly increased by DEX (*P*<0.05, Figure 3). FA had a significant effect on plasma glucose (*P*<0.05), insulin (*P*<0.05), TG (*P*<0.05), and VLDL (*P*<0.0001). PA chickens had higher levels of glucose, insulin, and VLDL than those in the OA and PLA groups, and TG concentration was increased after PA and OA infusion compared with PLA treatment. DEX and FA interacted for VLDL (*P*<0.001) but not for glucose, insulin, and TG. For the DEX treated chickens, the stimulating effect of DEX on VLDL concentration was enhanced by PA and alleviated by OA infusion.

### Enzyme Activity

**Figure 4:**
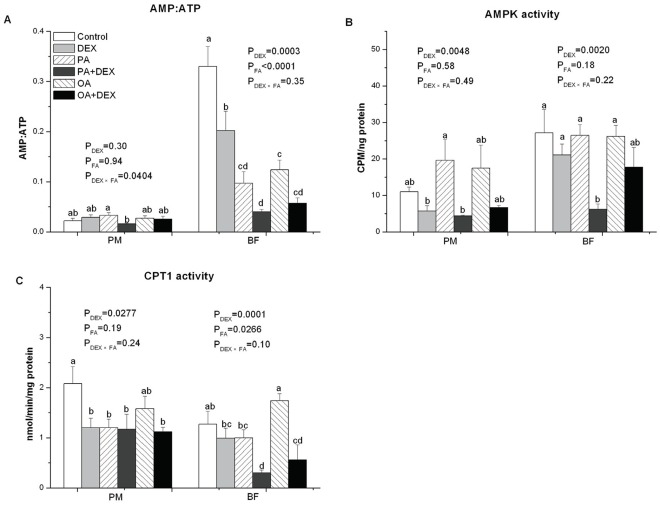
The effect of DEX and FA on AMPK/CPT signal pathway. Figure A–C show the effect of DEX (daily subcutaneous injection of 2 mg/kg body mass for 3d) and FA treatments (daily gastric infusion of either palmitic acid or oleic acid for 7d) and the interaction between DEX and FA on AMP to ATP ratio (A), AMPK activity (B) and CPT1 activity (C) in PM and BF of broiler chickens. The values are the means ± SE (n = 8); Means with a different letter differ significantly (*P*<0.05). P_DEX_: P-value of main effect of DEX treatment; P_FA_: P-value of main effect of FA treatment; P_DEX×FA_: P-value of interaction between DEX and FA treatments.

In PM, neither DEX nor FA significantly affected the AMP to ATP ratio (*P*<0.05, Figure 4A). However, there was a significant interaction between DEX and FA treatments (*P*<0.05), and the DEX-PA chickens had a lower AMP to ATP ratio than the PA group. In contrast, both DEX and FA treatments had a significant effect on the AMP to ATP ratio in BF (*P*<0.001). DEX treated chickens had a significantly lower AMP to ATP ratio than NORM chickens. Compared with the PLA group, the AMP to ATP ratio was decreased after PA and OA treatment.

The AMPK activity was significantly suppressed by DEX in both PM and BF tissues (*P*<0.01, Figure 4B). FA treatment, however, had no significant influence on AMPK activity in either PM or BF and no significant interaction. CPT1 activity was suppressed by DEX treatment in PM (*P*<0.05) and BF muscle (*P*<0.001, Figure 4C). In contrast, FA treatment affected significantly only in BF, and CPT1 activity was decreased by PA infusion (*P*<0.05).

### Gene mRNA Expression

**Figure 5:**
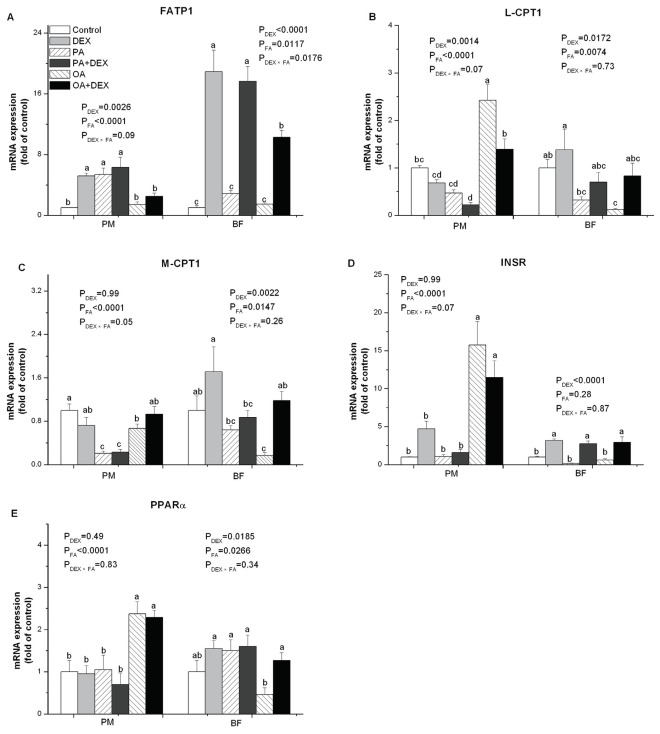
The effect of DEX and FA on lipid-related gene mRNA expression. Figure A−E show the effect of DEX (daily subcutaneous injection of 2 mg/kg body mass for 3d) and FA treatments (daily gastric infusion of either palmitic acid or oleic acid for 7d) and the interaction between DEX and FA on the mRNA expression of FATP1 (A), L-CPT1 (B), M-CPT1 (C), INSR (D), and PPARα (E) in PM and BF of broiler chickens. The values are the means ± SE (n = 6); Means with a different letter differ significantly (*P*<0.05). P_DEX_: P-value of main effect of DEX treatment; P_FA_: P-value of main effect of FA treatment; P_DEX×FA_: P-value of interaction between DEX and FA treatments.

DEX treatment significantly upregulated the gene expression of fatty acid transport protein 1 (FATP1) in PM (*P*<0.01) and in BF (*P*<0.0001, Figure 5A). In contrast, the FATP1 mRNA level was significantly increased by PA in PM (*P*<0.0001) and decreased by OA in BF (*P*<0.05), compared with PLA-treated chickens. DEX and FA interacted significantly in BF (*P*<0.05), and the effect from DEX was suppressed by OA infusion.

DEX treatment significantly downregulated the expression level of liver-carnitine palmitoyl transferase 1 (L-CPT1) in PM (*P*<0.01), whereas the converse was true in BF (*P*<0.05, Figure 5B). In contrast, muscle-carnitine palmitoyl transferase 1 (M-CPT1) expression differed between DEX and NORM only in BF (*P*<0.01) and not in PM (Figure 5C). The mRNA levels for L-CPT1 and M-CPT1 in PM were significantly decreased by PA, whereas OA infusion significantly upregulated the expression of L-CPT1 (*P*<0.0001). In BF, however, L-CPT1 and M-CPT1 expressions were significantly downregulated by either PA or OA (*P*<0.05).

DEX treatment had no significant influence on insulin receptor (INSR) gene expression in PM, but the converse was true for BF (*P*<0.0001, Figure 5D). In contrast, FA treatment had a significant effect on PM (*P*<0.0001) but not BF. The INSR mRNA level was significantly upregulated by OA infusion compared with the PA and PLA groups in PM.

PPARα gene expression was significantly upregulated by DEX in BF (*P*<0.05) but not in PM (Figure 5E). FA treatment significantly influenced the PPARα mRNA level in PM (*P*<0.0001) and BF (*P*<0.05). The PPARα expression level was upregulated by OA in PM but was downregulated by OA in BF.

### Protein Phosphorylation

**Figure 6:**
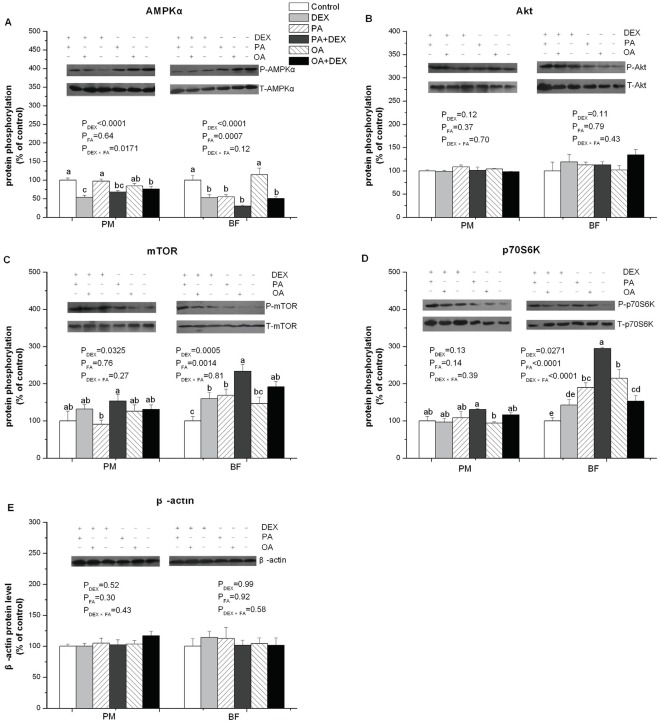
The effect of DEX and FA on lipid-related gene protein expression. Figure A−E show the effect of DEX (daily subcutaneous injection of 2 mg/kg body mass for 3d) and FA treatments (daily gastric infusion of either palmitic acid or oleic acid for 7d) and the interaction between DEX and FA on the protein phosphorylation of AMPKα (A), Akt (B), mTOR (C), and p70S6K (D), as well as the β-actin protein level (E) in PM and BF of broiler chickens. The values are the means ± SE (n = 4); Means with a different letter differ significantly (*P*<0.05). P_DEX_: P-value of main effect of DEX treatment; P_FA_: P-value of main effect of FA treatment; P_DEX×FA_: P-value of interaction between DEX and FA treatments.

As an internal control, β-actin protein abundance was not significantly affected by either DEX, FA, or an interaction between treatments in either PM or BF (Figure 6E).

The abundance of the P-AMPKα protein at Thr172 was significantly decreased by DEX in PM and BF (*P*<0.0001, Figure 6A). In contrast, a significant effect from FA treatment was detected in BF (*P*<0.001) but not PM. In BF, PA decreased the P-AMPKα level compared with either the OA or PLA group. DEX and FA treatments interacted in PM (*P*<0.05). In the DEX treated chickens, the OA chickens had higher P-AMPKα level compared with the DEX-PLA chickens.

The phosphorylation level of Akt at Ser473 was not affected by either DEX or FA treatment (Figure 6B). DEX treatment significantly suppressed the phosphorylation level of mTOR at Ser2448 in PM (*P*<0.05) and BF (*P*<0.001, Figure 6C). A significant effect from FA treatment was detected only in BF, and the phosphorylation of mTOR was higher after PA and OA treatments than in the PLA group (*P*<0.01). In PM, neither DEX nor FA treatment had a significant effect on the phosphorylation level of p70S6K at Thr389 (Figure 6D). In contrast, in BF, DEX treatment increased the level of P-p70S6K (*P*<0.05). Compared with the PLA group, PA or OA infusion significantly upregulated the level of phosphorylated p70S6K (*P*<0.0001). DEX and FA treatments interacted in BF (*P*<0.0001). After DEX treatment, the PA chickens had higher P-p70S6K level compared with the DEX-PLA chickens.

## Discussion

We firstly demonstrated the interaction between GC and FA treatments on IMF accumulation in broiler chickens. The present study shows that exogenous GC administration and dietary PA infusion augmented the IMF content in skeletal muscle tissues by improving fatty acid uptake and suppressing fatty acid oxidation. DEX-enhanced IMF accumulation was aggravated by SFA (PA) but alleviated by UFA (OA) infusion. The result suggests that suppressed AMPK and augmented mTOR signaling pathways may be involved in enhanced IMF accumulation by GC.

### DEX Facilitates IMF Accumulation

In previous studies, GC administration increased hepatic lipogenesis and fat accumulation in adipose tissues in rats [40] and chickens [41]. Consistent with previous results, DEX treatment retarded BM gain, increased liver weight, and abdominal fat in chickens, which suggests an altered energy redistribution toward lipid deposition. Likewise, enhanced IMF accumulation by DEX exposure was consistent with previous result in rats [42] and suggested that GC augmented IMF in chickens. Moreover, DEX increased IMF content in PM and BF, indicating that DEX has an effect regardless of muscle types.

Lipid accumulation in skeletal muscle cells primarily depends on the net balance between fatty acid uptake and utilization. In this process, the genes related to fatty acid transport and oxidation, such as FATP1 and CPT1, are involved [3]. In DEX-treated chickens, FATP1 overexpression in PM (1.70 fold of NORM) and BF (8.96 fold of NORM) suggests an increased fatty acid uptake capacity in skeletal muscles, especially in BF, and the increased plasma TG and VLDL concentrations indicate an augmented circulating lipid flux. Moreover, the insulin-stimulated lipid uptake by skeletal muscle was reduced in FATP1-null rats [43], demonstrating that insulin can regulate fatty acid uptake via FATP1 activation, and INSR expression herein was upregulated in response to the increased circulating insulin. These results suggest that DEX treatment improves fatty acid uptake in skeletal muscles.

In rats, it was speculated that the relative increase in IMF after DEX treatment resulted from a decrease in lipid oxidation [42], and the suppression in conversion of inactive to active cortisol improved lipid oxidation in oxidative muscle [44]. Consistent with these reports, herein, the decreased CPT1 activity by DEX in both muscles indicates suppressed lipid oxidation. Moreover, the transcription of L-CPT1, but not M-CPT1, was attenuated in PM, while L-CPT1 and M-CPT1 expression were upregulated in BF, which indicates that the effect of DEX on the regulation of CPT1 is muscle-type dependent.

Collectively, the results suggest that excessive GC triggers IMF accumulation, primarily through the facilitated fatty acid uptake and retarded fatty acid oxidation in either glycolytic or oxidative muscle.

### AMPK Inhibition and mTOR Activation are Involved in DEX-facilitated IMF Accumulation

It is thought that AMPK is an important regulator of intracellular fatty acid metabolism. Phosphorylation at Thr172 in an α isoform by an upstream kinase is known to be required for AMPK activity [45]. In rats, acute DEX administration promoted ACC phosphorylation and increased cardiac PA oxidation, likely by activating AMPK [46], and chronic GC treatment inhibited AMPK activity in adipose tissue and heart, while stimulating it in the liver and hypothalamus [47]. Recently, it was reported that transcriptomic analysis of the liver suggested marked overlap between the AMPK and GC signaling pathways that was primarily directed by AMPK to GC action through an AMPK-mediated energy control system that modulates GC action at target tissues [48]. In the chicken herein, the lower AMP to ATP ratio coincided with decreased AMPK activity and AMPKα phosphorylation in BF, and the decreased CPT1 activity was consistent with the observation that the AMPK activity and phosphorylation were suppressed, which suggests that the AMPK signaling pathway is involved in the suppressed fatty acid oxidation in DEX-treated chickens. In a rat model, AMPK activity in response to muscle electrical stimulation was reduced after a 2-wk treatment of GC, and this response is believed to be involved in insulin resistance and adiposity [49]. This result suggests that the AMPK pathway is suppressed by long-term DEX administration, which is likely responsible, at least partially, for the augmented IMF accumulation.

In mammals, there is evidence to demonstrate the linkage of mTOR signaling with lipid metabolism. Um et al. [50] reported that S6K1-deficient mice exhibited an elevated CPT1 mRNA expression and an enhanced fatty acid oxidation capacity in skeletal muscle. Sipula et al. [18] reported that physiological mTOR inhibition promoted β-oxidation, at least in part, as a direct result of CPT activity increase, indicating a role for mTOR downregulation in lipid utilization. Acute DEX treatment suppressed p70S6K phosphorylation [20]. In rats, chronic DEX treatment abrogated the stimulatory effect of amino acid infusion on the phosphorylation of 4E-BP1, without affecting p70S6K [51]. In the present study, DEX upregulated the P-mTOR level in both muscles as well as the P-p70S6K level in BF, indicating that DEX treatment activated the mTOR pathway. The result suggests that the mTOR signaling pathway may be involved in GC regulation of muscle lipid metabolism. These novel observations in chickens are consistent with the results of Um et al. (50) and Sipula et al. [18] in mammals, who reported that mTOR inhibition was shown to elevate CPT1 mRNA expression and enhance muscle fatty acid oxidation. Phosphorylation of mTOR Ser2448, which positively correlated with mTOR activity [52], was inhibited by AMPK activation [53]. As upstream physiological counter-regulators of mTOR, lipid-induced mTOR activation and insulin resistance were restored to normal by activated AMPK in both *in vivo* rat skeletal muscle [19] and hepatocytes [54]. The crosstalk among the GC, AMPK, and mTOR signaling pathways must be investigated further.

Insulin and downstream signaling proteins, such as phosphatidylinositol-3-kinase (PI3K) and Akt, play an important role in glucose and lipid metabolism. Although there is an apparent insulin insensitivity in chicken muscle, GC may impair insulin signaling in chicken muscle to some extent [55], through competing with insulin receptor substrate 1 for association of PI3K [56]. In this study, DEX administration resulted in insulin resistance, as indicated by hyperglycemia and hyperinsulinemia, which is consistent with previous results in mammals [57] and chickens [23]. Akt expression was not affected by DEX treatment, even with hyperinsulinemia and upregulated INSR, indicating the unaltered insulin signaling cascade. In chickens, however, the early steps of the insulin signaling pathway differ from those of mammals, basal levels of tyrosine phosphorylation of INSR and of PI3K activity are much higher in chickens than in rats [4]. Contrasting with the strong activation of all components of the cascade by insulin in rats, none was observed in chicken muscles [4], [55]. GC impairs insulin signaling in response to re-feeding to some extent in chicken muscle (55), and the present result demonstrates that GC does not impair the insulin signaling pathway at basal state. In chickens, the Akt/TOR/p70S6K pathway is activated by re-feeding and insulin injection [58]. The activated mTOR signals in DEX-treated chickens in this study imply that DEX can induce the mTOR signaling pathway via other pathways. The role of Akt in GC-mediated lipid metabolism needs a further study *in vitro*.

In humans, skeletal muscle PPARα expression and genes regulating lipid metabolism are tightly linked [59]. Consistent with this result, the present results showed a similar trend in the gene expression of M-CPT1 and PPARα in DEX-treated chickens. PPARα activation increased fatty acid oxidation in skeletal muscle [60], and AMPK activation increased skeletal muscle lipid oxidation by activating PPARα [14]. Herein, the PPARα expression was upregulated in BF and unaffected in PM, suggesting that the change in the PPARα gene expression does not play an important role in DEX-altered lipid metabolism.

Collectively, these results suggest that suppressed AMPK and activated mTOR signals are involved in DEX-induced IMF accumulation.

### IMF Accumulation is Aggravated by PA Rather than OA

Although UFA and SFA can induce insulin resistance and obesity, there is evidence to suggest that SFA is more effective than UFA [61], [62]. PA and OA are controlled differently by myotubes. PA accumulates as diacylglycerol and triacylglycerol, whereas OA accumulates as intracellular free fatty acid [63], which indicates that the OA is preferable for oxidation. Moreover, SFA, but not UFA, promoted ceramide accumulation, which is a common molecular intermediate linking several different pathological metabolic stresses to the induction of insulin resistance [64]. In accordance with previous results in mammals, the IMF content augmented by PA, not OA, indicates that PA facilitates IMF accumulation in chickens. Coinciding with increased IMF content from PA treatment, blood glucose and lipid flux were increased in PA-treated chickens (glucose, +6.06%; VLDL, +14.9%) but the change was less obvious from OA infusion (glucose, −1.23%; VLDL, +10.6%).

The upregulated FATP1 gene expression in PM, downregulated L- and M-CPT1 gene expression and suppressed CPT1 activity by PA infusion in PM and BF indicate that PA infusion induces lipid accumulation and suppresses lipid oxidation. In contrast, the decreased FATP1 expression and unaffected CPT1 activity in BF from OA treatment should be responsible, at least partially, for the unaffected IMF content.

Hickson-Bick et al. [65] reported that AMPK was activated in cultured rat cardiomyocytes by PA and OA treatment. AMPKα Thr172 was also phosphorylated in rat hearts infused with SFA without changes to cellular energy charge [66]. Further, SFA and UFA increased AMPK activity independent of changes in cellular AMP content in L6 myoblasts [67]. These results imply that increased fatty acid availability may promote AMPK activation, independent of the cellular AMP level. Herein, AMPK activity was not significantly affected by FA treatment in either muscles, and AMPKα phosphorylation was decreased in BF by PA infusion. This result suggests that AMPK activity may not play a primary role in increased IMF accumulation after PA treatment.

Recent evidence demonstrates that muscle mTOR is overactivated in obese rodents, high-fat-fed rodents and *in vitro* L6 myotubes exposed to PA [19], [68], [69], and S6K1-null mice are protected from high fat diet-induced adverse effects [50]. In accordance with the studies in rodents, increased mTOR and p70S6K phosphorylation in BF indicates the activation of mTOR signals by FA infusion in chicken oxidative skeletal muscles. In the glycolytic muscle, however, mTOR and p70S6K phosphorylation were unaffected by FA treatment, suggesting that the effect of FA treatment on the mTOR signaling pathway is tissue specific.

One of the major sites for SFA-mediated adverse function is INSR [70]. In contrast, OA improves insulin signaling and protects against PA-induced insulin resistance in L6 myotubes [71]. We also observed that OA upregulated INSR mRNA expression in glycolytic muscle and did not significantly alter the circulating glucose/insulin balance, which indicates enhanced insulin sensitivity. Akt Ser473 phosphorylation was significantly increased in L6 myotubes after PA incubation [19], in contrast, PA treatment had no effects on INSR expression or Akt phosphorylation in present result. The PA CoA ester formation is critical for its adverse effect on insulin action in hepatocyte cell [70], thus the *in vivo* effect of FA treatment on the insulin signaling must be investigated further.

Recently, PPARα signaling was shown to be associated with the regulation of optimal substrate selection and disposition in skeletal muscle according to its metabolic requirement [72]. Staiger et al. [9] reported that PA and OA regulated PPARs expression differentially, which suggests that mitochondrial activity was increased by UFA but suppressed by SFA. The result herein indicates that the regulation of FA on PPARα expression is dependent on muscle type.

### DEX-enhanced IMF Accumulation is Aggravated by PA, but Alleviated by OA

The stress response is interfered by a high-fat diet in rat model [73]. The combination of social stress and high-fat diet in a mice model altered energy portioning with increased body fat [74]. Excess GC aggravated the development of glucose intolerance in rats fed a high-fat diet [75]. Recently, it was proved that GC and SFA were linked by ceramide to the induction of insulin resistance in rats [64]. Herein, consistent with the previous studies in mammals, there was also an interaction between the DEX and FA treatments in chicken model. This finding could allow a novel view on different biological systems. We found that the DEX-stimulated IMF deposition and plasma VLDL concentration were aggravated by PA but alleviated by OA, and several pathways including AMPK and p70S6K may be involved. The OA infusion alleviated the DEX-decreased AMPKα.phosphorylation in PM, and PA treatment accelerated the stimulating effect of DEX on P-p70S6K level in BF. Compared with UFA enriched diet, SFA stimulated conversion of inactive to active GC in rats [76], which could explain the different regulation of PA and OA on DEX action in present study.

Given the function of FATP1 and CPT1 on fatty acid uptake and oxidation respectively, we speculate that the interaction between DEX and FA treatments on FATP1 and CPT1 may contribute to the altered IMF accumulation. The results showed that the stimulating effect of DEX on FATP1 expression was suppressed by OA infusion. Moreover, the stimulating effect of OA on L-CPT1 expression in PM was alleviated in the presence of DEX (*P* = 0.07), and the suppression effect of OA on M-CPT1 in PM was not detected in DEX-treated chickens (*P* = 0.06). However, the change at the transcriptional level was not consistent with the observations on CPT1 activity, which suggests that OA interacts with DEX only at the mRNA level.

In conclusion, DEX facilitated intramyocellular lipid accumulation by incompatible fatty acid uptake and utilization. DEX-induced IMF deposition was aggravated by SFA but alleviated by UFA. This result suggests that suppressed AMPK and augmented mTOR signaling pathways are involved. The regulating pathways associated with *in vivo* FA infusion must be investigated further.

## Supporting Information

Figure S1 Institutional Animal Care and Use Committee of Shandong Agricultural University.
(TIF)
