# Supplementary figures and images for: Effects of Fatty Acid Treatments on the Dexamethasone-Induced Intramuscular Lipid Accumulation in Chickens
Source: PLoS One. 2012 May 18;7(5):e36663. doi: 10.1371/journal.pone.0036663 (PMC3356436; doi:10.1371/journal.pone.0036663)

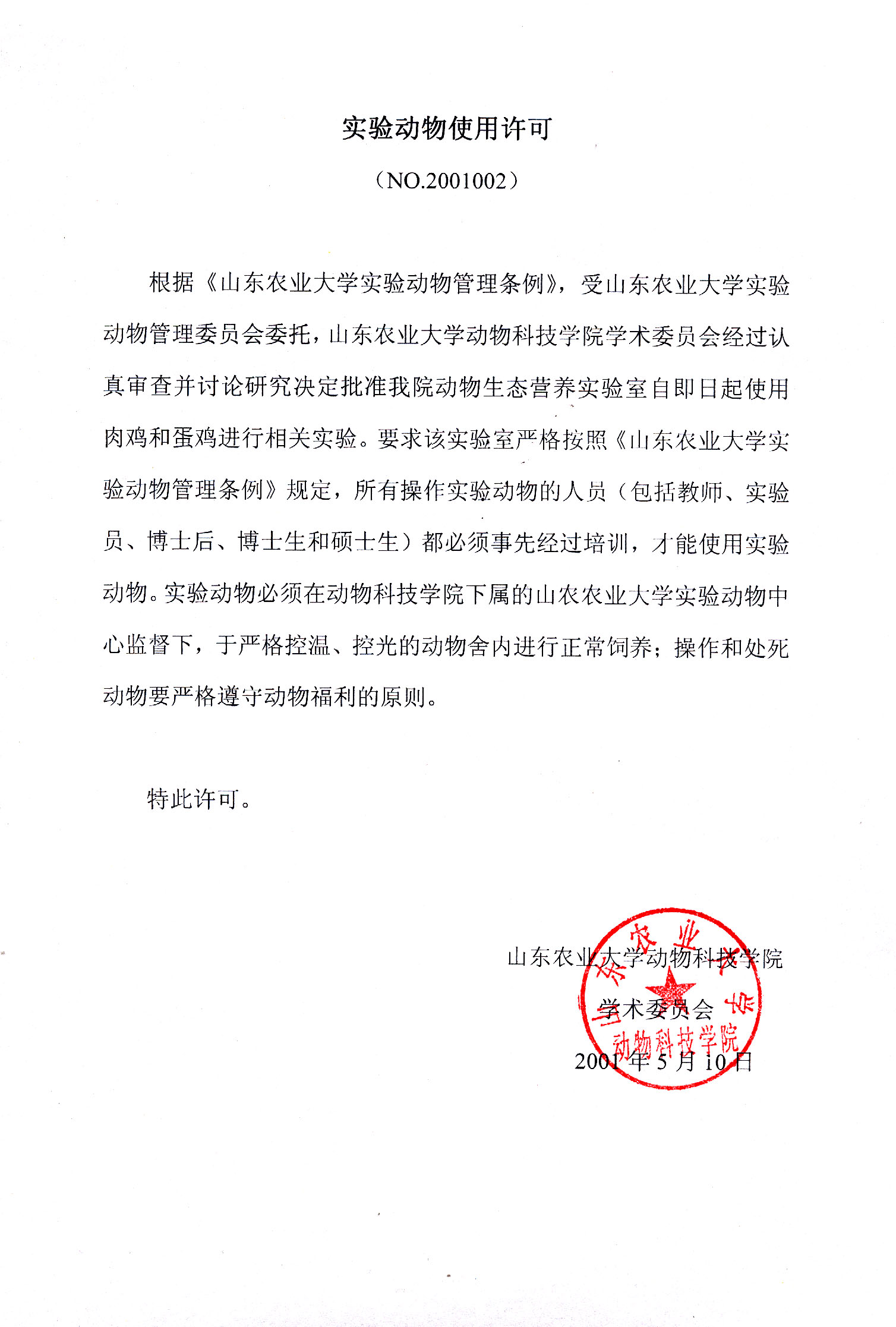

Supplement: Figure S1 — Institutional Animal Care and Use Committee of Shandong Agricultural University. (TIF) [file pone.0036663.s001.tif]
